# Bacterial vaginosis and the gut-vagina axis

**DOI:** 10.1128/spectrum.01244-25

**Published:** 2025-07-31

**Authors:** Kazuhide Takada, Shihoko Komine-Aizawa

**Affiliations:** 1 Division of Microbiology, Department of Pathology and Microbiology, Nihon University School of Medicine 38113, Tokyo, Japan; Chengdu University, Chengdu, Sichuan, China.

**Keywords:** vaginitis, vaginosis, microbiota

## LETTER

We read with great interest the study by Li et al. (1). In this study, the authors performed a Mendelian randomization analysis and identified *Candidatus Soleaferrea* and *Dialister* in the gut as potential risk factors for vaginitis, while *Lachnospiraceae UCG-008* and *Ruminiclostridium 5* were negatively associated with vaginitis. They also demonstrated that the stimulating effects of *C. Soleaferrea* and *Dialister* in vaginitis were antagonized by the plasma metabolites 3-phosphoglycerate and lysophosphatidylcholine, respectively. In contrast, the protective effects of *Lachnospiraceae* against vaginitis were enhanced by a decreased palmitate/myristate ratio and an increased arachidonate/pyruvate ratio.

In their study, the authors used the GWAS Catalog Database (ID: GCST90044303) to perform their analysis and adhered to the International Classification of Diseases (10th edition) definition of vaginitis. However, the term “vaginitis” requires careful consideration. Vaginitis is a general term referring to vaginal infections with a variety of etiologies, including vulvovaginal candidiasis (17%–39%), bacterial vaginosis (BV) (22%–50%), and trichomoniasis (4%–35%) (2). The presence of a specific pathogen typically leads to the naming of the condition after the infectious agent identified as responsible for the symptoms, such as candidiasis or trichomoniasis. In contrast, the dysbiosis in BV is characterized by a shift in the vaginal microbiota from a *Lactobacillus*-dominated one to one dominated by facultative or obligate anaerobes (3), which makes it more difficult to provide meaningful and descriptive terminology (4). The initially characterized syndrome of vaginitis encompassed a range of mucosal inflammation-related symptoms, such as vaginal discharge, itching, and burning. However, BV was noted for the absence of a leukocytic exudate, redness, and swelling. Consequently, to differentiate it from traditional vaginitis, the term “vaginosis” was coined (4). In other words, unlike other types of vaginitis that are triggered by specific microorganisms, BV frequently manifests without the typical signs of an acute infectious inflammatory process that would normally involve elevated levels of polymorphonuclear cells in the vaginal discharge (4). The traditional definition of vaginitis used in this study thus encompasses a number of different pathophysiological disorders.

Recent studies have shown that BV is not a distinct microbiological condition but rather a general term encompassing clinical signs and symptoms resulting from inflammation caused by various bacterial species (4). Thus, BV remains difficult to define and characterize using conventional diagnostic approaches. Although the new approach by Li et al. (1) utilizing a Mendelian randomization analysis provides intriguing evidence that gut microbiota may contribute to the development of vaginitis, we suggest that future analyses should stratify cases based on their underlying etiology. This stratified approach may yield more detailed insights into the pathogenesis of BV, particularly in the aspect of the gut–vagina axis (5).

## EDITOR'S NOTE

**Ed. Note:** The authors of the original article did not respond at the time of publication
